# The Detrimental Effect of Sexual Objectification on Targets’ and Perpetrators’ Sexual Satisfaction: The Mediating Role of Sexual Coercion

**DOI:** 10.3389/fpsyg.2019.02748

**Published:** 2019-12-11

**Authors:** Gemma Sáez, María Alonso-Ferres, Marta Garrido-Macías, Inmaculada Valor-Segura, Francisca Expósito

**Affiliations:** ^1^Departamento de Psicología, Universidad Loyola Andalucía, Seville, Spain; ^2^Department of Social Psychology, Faculty of Psychology, Mind, Brain and Behavioral Research Center (CIMCYC), University of Granada, Granada, Spain

**Keywords:** sexual objectification, sexual coercion, sexual satisfaction, unwanted sexual rejection, gender differences

## Abstract

Sexual objectification is a variable to consider for understanding the sexual violence that takes place into intimate context. The set of studies presented here aims to connect sexual objectification phenomena with sexual coercion and explore the consequences that both have on sexual satisfaction. Two studies examined the association between sexual objectification and sexual satisfaction for both views: female target (Study 1) and male perpetrator (Study 2) perspectives. The results of the first study (*n* = 138 heterosexual women) demonstrated that perceiving partner objectification (but not reporting general sexual objectification victimization) is indirectly linked to a lower sexual satisfaction because of lower rejection and higher sexual coercion rates. The second study (*n* = 136 heterosexual men) showed the indirect effect of partner objectification and general sexual objectification perpetration on sexual satisfaction after sexual coercion perpetration. Results of both studies demonstrated the negative consequences that sexual objectification has on sexual satisfaction for both male perpetrators and female targets.

## Introduction

### Sexual Objectification

Objectification Theory (Fredrickson and Roberts, 1997) was developed for understanding the negative consequences that result from living in a culture that sexually objectifies women. Specifically, sexual objectification and its internalization (self-objectification—described as internalization of observers’ perspective of their own bodies, which result in valuing how their own bodies look ignoring how their bodies act—Fredrickson and Roberts, 1997; Roberts et al., 2018) has showed to have detrimental consequences for women in an intrapersonal (e.g., eating disorders and depression: Tiggemann and Kuring, 2004), interpersonal (sexual dissatisfaction and relationship dissatisfaction: Zurbriggen et al., 2011), and social level (Saguy et al., 2010; Sáez et al., 2012). Indeed, more than 20 years of research have showed the negative consequences of self-objectification on women’s health, motivations, affections, and cognitive, behavioral, and social spheres (for a review, see Roberts et al., 2018).

Sexual objectification is a gendered phenomenon, which involves victims (mostly women) and perpetrators (mostly men; Strelan and Hargreaves, 2005; Gervais et al., 2018). However, it has negative consequences for women (e.g., Calogero and Thompson, 2009; Sáez et al., 2012) and men (Wright, 2011). Even though feminist scholars have theorized that objectification within a romantic relationship might be a natural component of a sexual relationship (Nussbaum, 1999), we propose that sexual objectification could also have detrimental consequences for individuals’ sexual satisfaction (e.g., Steer and Tiggemann, 2008).

### Female Sexual Objectification and Sexual Coercion Victimization and Their Consequences for Female Sexual Satisfaction

Objectification Theory proposes that self-objectification, as a consequence of being the target of sexually objectifying experiences, leads to female sexual dysfunction, among other health negative consequences (Fredrickson and Roberts, 1997). Empirical research has given support to the Objectification Theory, showing that self-objectification predicts lower sexual satisfaction (Calogero and Thompson, 2009) and lower sexual functioning (Steer and Tiggemann, 2008). Specifically, Calogero and Thompson (2009) showed that internalization of appearance ideals leads to greater surveillance and body shame, leading to lower sexual self-esteem, which turns into lower sexual dissatisfaction. Moreover, the negative effect of sexual objectification on sexual satisfaction might be alternatively explained by self-consciousness during sexual intercourse having a negative effect on sexual satisfaction (Claudat and Warren, 2014). In addition to the indirect effect of sexual objectification on lower sexual satisfaction because of self-objectification, being treated as a sexual object on an interpersonal level is directly related to insidious trauma symptoms (Miles-McLean et al., 2015) and safety anxiety (Fairchild and Rudman, 2008), which might prevent women from enjoying their sexual life.

Regarding the romantic relationship context, scant studies have explored the effects of sexual objectification and its sexual outcomes. In a recent study, Sáez et al. (2019) showed that for women, perceiving their male partners as dehumanizing them is indirectly related to lower sexual satisfaction because the women have a damaged body image. Moreover, Ramsey and Hoyt (2015) showed that partner objectification negatively affects the female sexual agency. From the previous literature, conclusions might be drawn that being sexually objectified at the interpersonal or romantic level has negative consequences for female sexual satisfaction.

Correlational studies have showed that sexual objectification could be a key mechanism for understanding sexual victimization. For example, Davidson and Gervais (2015) showed that self-objectification was related to sexual violence victimization. Likewise, Haikalis et al. (2017) showed that the link between alcohol use (frequency and quantity) and sexual victimization was mediated and thus explained by sexual objectification. More importantly, sexual objectification experiences are negatively related to the refusal of sexual acts, showing that there is an indirect relationship between body evaluation experiences and lower sexual assertiveness in women specifically related to the refusal of unwanted sexual acts because of self-objectification (Franz et al., 2016). Franz et al. (2016) pointed out the positive relation between sexual objectification experiences and female vulnerability to sexual victimization, with the lower rejection of unwanted sexual advances as the identified mechanism.

Scant research has explored the effect of partner objectification on sexual assertiveness (for an exception, see Ramsey and Hoyt, 2015). Ramsey and Hoyt (2015) concluded that women who report their male partner’s surveillance of their body are less able to refuse sex from their partner. This result is especially relevant because women’s decreased ability to refuse sex increases their probability of suffering sexual victimization. Importantly, the perpetrators of sexual violence are solely responsible for such acts, but there are some female variables that have been related to an increase in their likelihood of suffering sexual assault. For example, assertiveness in refusing sexual activity is crucial for avoiding sexual assault (Gidycz et al., 2008; Wigderson and Katz, 2015). Women who have difficulty refusing sexual requests are more vulnerable to sexual victimization (e.g., Livingston et al., 2007; Katz et al., 2010; Santos-Iglesias and Sierra, 2012; Ullman and Vasquez, 2015; Wigderson and Katz, 2015).

Sexual assertiveness is especially important in cases of sexual coercion. Specifically, sexual coercion refers to any behavior carried out to make another person to participate unwillingly in vaginal, oral, or anal sex, through the use of verbal pressure, threat, or physical force (He et al., 2013; Bagwell-Gray et al., 2015; Smith et al., 2018; Garrido-Macías et al., in press). Given that sexual coercion occurs in the context of verbal and emotional pressure and is more frequent in a romantic relationship context, it is plausible that sexual assertiveness may play an influential role in cases of sexual coercion (Testa and Dermen, 1999; Walker et al., 2011). Walker et al. (2011) found that sexual assertiveness decreases the experiences of sexual coercion and Testa et al. (2007) showed that low assertiveness in sexual refusal was associated with sexual coercion and revictimization experiences in a romantic context.

Sexual coercion can have negative consequences for female victims in the form of sexually transmitted infections, lowered sexual self-esteem, increased sexual depression, negative sexual self-perceptions, and negative emotions (e.g., Zweig et al., 1999; Offman and Matheson, 2004; Shackelford and Goetz, 2004; Garrido-Macías et al., 2017). One of the well-known negative consequences of sexual victimization is sexual dissatisfaction (Bartoi and Kinder, 1998; Katz and Myhr, 2008). Specifically, Katz and Myhr (2008), using a sample of 193 college women in consensually sexual dating relationships, showed that women who had experienced sexual coercion by their partners also reported lower sexual satisfaction and lower sexual desire than women who had not been victims of sexual coercion.

### Male Sexual Objectification and Sexual Coercion Perpetration and Their Consequences for Male Sexual Satisfaction

Despite the fact that objectification theory was developed for understanding the negative consequences that sexual objectification has on women’s well-being (Fredrickson and Roberts, 1997), empirical research in this field has overtaken the original assumptions, showing that sexual objectification has detrimental consequences for the men who perpetrate it, too. Men’s objectifying lens influences their thoughts and behaviors (Gervais et al., 2018). Elder et al. (2012, p. 170) showed a high prevalence of sexual objectification perpetration by evidencing in their qualitative study that male participants frequently reported “looking at,” “watching,” “viewing,” and “checking out” female bodies. Importantly, sexual objectification perpetration has been related to sexual violence (Gervais et al., 2014) and could be considered a behavioral predictor of an extreme sexual violence manifestation, or in the words of Haikalis et al. (2017), “Commonplace forms of objectification may play as potential contributors to full-fledged sexual assault” (p. 17). Moreover, Rudman and Mescher (2012), showed that objectifying perception of women predicts rape proclivity; when men implicitly objectifying women, they are more likely to sexually victimize them.

Sexual objectification has been thought of as a natural component of the sexual relationship (Nussbaum, 1999) based on the fact that sexual attraction is positively related to sexual relationship satisfaction (Galdi et al., 2014). However, empirical research shows that men’s objectification of their romantic partner has detrimental effects on male sexual satisfaction (Zurbriggen et al., 2011). Authors asserted that considering and treating female partners as objects hinder men to create personal and emotional connection with them, which turn on lower levels of sexual satisfaction (Zurbriggen et al., 2011). A clear manifestation of these assumptions could be found in pornography consumption—where women are literally reduced to sex objects. For example, previous studies showed that men who watch more pornography tend to treat women as objects (Mikorski and Szymanski, 2017), endorse objectified cognitions about them (Wright and Tokunaga, 2015), and therefore, to see their sexual satisfaction reduced (Zillmann and Bryant, 1988).

In addition to objectification literature, Centerfold Syndrome (Brooks, 1995), which is defined as a set of beliefs about the sexuality of heterosexual men (voyeurism, sexual reductionism, masculinity validation, trophyism, and nonrelational sex), has articulated the negative effect of objectification media exposure on interpersonal relationships. The Centerfold Syndrome hypothesis articulates the negative effect of objectification media exposure on interpersonal relationships. Specifically, Wright and Tokunaga (2015) showed that past objectifying media exposure lead to centerfold beliefs among men. This theory asserts that men who hold centerfold beliefs have more difficulties in establishing an intimate relationship with women. Sexual objectification socialization makes establishing healthy intimate and sexual relationships with women difficult for men because the objectifying media gives men unrealistic expectations about their female partners’ appearance and leads to lower satisfaction in the intimate domain (Brooks, 1995; Wright, 2012). Beyond objectifying media, the high occurrence of male objectifying behaviors (gazing, commenting, or making unwanted sexual advances) leads to negative outcomes in their intimate relationships (Sáez et al., 2019).

One of the most adverse sexual objectification outcomes on an intimate domain might be sexually aggressive treatment of women. Treating women as objects means using them as tools for satisfying their male partner’s sexual desires (Bartky, 1990), which might lead to sexual coercion of women. Based on that, our hypothesis is that men who generally sexually objectify women have a higher likelihood of sexually coercing their partner, being sexual objectification a potential risk factor for sexual coercion offenders. Previous research showed that heterosexual men who objectify their partners are more likely to sexually pressure and coerce their female partners in an intimate domain (Ramsey and Hoyt, 2015). Specifically, Ramsey and Hoyt (2015) showed that men’s chronic surveillance of their female partner’s body and thinking frequently about their female partner appearance is related to a higher rate of sexual coercion. The objectifying lens hinders men from having authentic and equal relationships with their female partners, leading the men to ignore their partner’s willingness in the sexual context.

Although it is evident that victims of sexual coercion have more negative consequences, the male aggressors also experience negative emotions, especially in their relationship satisfaction. Generally, literature has demonstrated that intimate partner violence (including both physical and psychological violence) is related to dissatisfaction (e.g., Stith et al., 2008; Hammett et al., 2017). Despite the negative effects of sexual aggression on relationship satisfaction, there are unknown studies that analyze the association between sexual coercion perpetration and sexual satisfaction. Therefore, we aim to overcome this gap in the literature by showing that sexual coercion is negatively related to sexual satisfaction in male perpetrators.

### The Current Research

Our general aim is to explore the role of sexual objectification in sexual satisfaction after sexual coercion perpetration and victimization. Specifically, we aim to explore the negative and indirect effect of sexual objectification on sexual satisfaction through sexual coercion. We hypothesized that the instrumentalization involved on female sexual objectification lead to higher use of sexual coercion strategies, which lastly will turn on lower female and male sexual satisfaction. By testing this effect among men perpetrators and women victims, it will help us to understanding why sexual objectification hinders sexual satisfaction. Moreover, by comparing the model predicted by partner objectification (victimization or perpetration) with the model predicted by general objectification (victimization or perpetration), we will determine what variable predicts in higher extent the well-known sexual objectification negative outcomes. With this aim, we ran two correlational studies assuming complementary gender perspectives.

In Study 1, the two main aims were to test whether interpersonal sexual objectification experiences (first aim) and partner objectification (second aim) have a detrimental effect on women’s capacity to reject sexual relationships, which will turn on more sexual coercion experiences and lower sexual satisfaction. Using a sample of female college students, we examined five hypotheses. First, we hypothesized that interpersonal sexual objectification experiences (Hypothesis 1a) and partner objectification (Hypothesis 1b) would have a negative effect on sexual satisfaction. Second, we hypothesized women’s experiences of interpersonal sexual objectification (Hypothesis 2a) and partner objectification (Hypothesis 2b) will be negatively related to their capacity for rejecting sexual intercourse. Third, we hypothesized that a lower rejection capacity will predict sexual coercion victimization (Hypothesis 3), which will lead to lower sexual satisfaction (Hypothesis 4). Lastly, we hypothesized an indirect effect of experiencing sexual objectification (Hypothesis 5a) and partner objectification (Hypothesis 5b) on sexual satisfaction through lower rejection and higher sexual coercion rates.

In Study 2, we take the perpetrators’ perspective by examining the relationships between the perpetrations of objectification of women in general and of their specific partner and the sexual satisfaction of men. With this aim and using a sample of male college students to test four hypotheses, first, we hypothesized that men’s objectification of women generally (Hypothesis 1a) and toward their specific partner (Hypothesis 1b) would predict decreased male sexual satisfaction. Moreover, we hypothesized that the frequency of sexual objectification of women in general (Hypothesis 2a) or of their specific partner (Hypothesis 2b) will predict sexually coercive behaviors toward the partner, which will be negatively related to sexual satisfaction (Hypothesis 3). Lastly, we tested a path analysis of men’s general perpetration of sexual objectification (Hypothesis 4a) or partner objectification (Hypothesis 4b) through a higher frequency of sexually coercive behaviors toward their partner.

## Study 1

### Method

#### Participants

A total of 155 women participated in the study. Of these, 12 were excluded from analyses because they did not identify as heterosexual and five because they reported to be single and had never been in a sexual relationship with their current or former partner. The remaining 138 participants ranged in age from 16 to 37 years old (*M* = 21.80, SD = 3.59). In the sample, a total of 57.6% of the participants were dating, 8.6% were cohabiting, and 0.7% were married, whereas 33.1% were not involved in a romantic relationship. Approximately 46.4% of the participants completed high school, and 53.6% were enrolled in a university degree program.

#### Procedure

Following an incidental sampling procedure in different university centers (e.g., libraries) of a Spanish city, two formed evaluators requested the participants’ collaboration. Specifically, evaluators informed the participants about the voluntary character of the study, the anonymity and confidentiality of their responses, and the study’s approximated duration. After that, participants were asked to complete the questionnaire survey individually and voluntarily. Additionally, single participants could answer the survey questions thinking about their most recent partner. After providing informed consent, participants completed the study surveys embedded within a larger series of questionnaires. All of the participants were volunteers and provided informed written consent, and no monetary incentives were provided for participation. Once the study was finished, participants were fully debriefed and thanked.

#### Measures

##### Interpersonal Sexual Objectification

To evaluate the extent to which participants felt objectified, participants completed the Interpersonal Sexual Objectification Scale (Lozano et al., 2015). This 15-item scale asks women to report on the frequency with which they receive a body evaluation (11 items; e.g., “How often have you felt that someone is staring at your body?”) and unwanted explicit sexual advances (four items; e.g., “How often have you been groped?”), using a Likert-type scale ranging from 1 (never) to 5 (almost always). Participants’ responses were averaged, with higher scores indicating more frequent objectification of women. The Interpersonal Sexual Objectification Scale demonstrated good internal consistency for the current sample (Cronbach’s *α* = 0.91).

##### Partner Objectification

In line with prior research (e.g., Ramsey and Hoyt, 2015), to assess how much a woman feels her partner surveys her body, we used a modified version of the body surveillance subscale of the Objectified Body Consciousness Scale (OBCS-P; Moya-Garófano et al., 2017). Participants were asked to evaluate the extent to which their partner monitors their body (e.g., “My partner often worries about whether the clothes I am wearing make me look good”) on a Likert-type scale ranging from 1 (disagree strongly) to 7 (agree strongly), with a “not applicable” option. Not applicable responses were coded as missing data and participants’ responses were averaged, with higher scores indicating greater objectification of their partner. The total partner objectification scale showed low reliability (= 0.59), so two items (5 and 6) with the lowest item-total correlations were excluded, resulting in a six-item scale with higher but still low reliability (= 0.60), similar to the alphas found in previous studies (Cronbach’s *α* = 0.71; Ramsey et al., 2017).

##### Sexual Advance Rejection

To evaluate the extent to which women were able to refuse unwanted sex, participants completed the Spanish version of the Sexual Assertiveness Scale (Sierra et al., 2011). This six-item subscale asks women to report on the frequency of which they have been able to refuse a sexual relationship (e.g., “I have sex if my partner wants me to, even if I don’t want to” reversed item), using a Likert-type scale ranging from 0 (never) to 4 (always). Participants’ responses were averaged, with higher scores indicating greater sexual rejection. The Sexual Assertiveness Scale demonstrated good internal consistency for the current sample (Cronbach’s *α* = 0.74).

##### Sexual Coercion Victimization

An abbreviated version composed by two subscales of the Sexual Coercion in Intimate Relationships Scale (SCIRS; Shackelford and Goetz, 2004) was used to assess sexual coercion victimization by an intimate partner. Participants indicated whether they had experienced 10 specific acts of *commitment manipulation* (e.g., “My partner hinted that if I loved him, I would have sex with him”), and nine specific acts of *defection threat* (e.g., “My partner hinted that he would have sex with another woman if I did not have sex with him”). Participants answered using a five-point response scale: 0 (never has occurred), 1 (has occurred once in the last month), 2 (has occurred twice in the last month), 3 (has occurred between three and five times in the last month), 4 (has occurred between six and 10 times in the last month), and 5 (has occurred more than 11 times in the last month). Full-scale scores were calculated by summing response values for each item in the entire scale, so that higher numbers indicated greater sexual coercion victimization. Because the item-level missing data can bias the sum score, we explored the percentage of the item-level missing data, and it was less than 5% of the participants, as Schafer (1999) suggested.

##### Sexual Satisfaction

The Global Measure of Sexual Satisfaction (GMSEX; Lawrance and Byers, 1995; Sánchez-Fuentes et al., 2015) was used to assess the extent to which participants were satisfied regarding their sexual relationship with their partner. Respondents rated their sexual satisfaction on seven-point bipolar scales (very bad-very good; very unpleasant-very pleasant; very negative-very positive; very unsatisfying-very satisfying; worthless-very valuable). Participants’ responses were summed (item-level missing data were less than 5%), with higher scores indicating greater sexual satisfaction. GMSEX demonstrated good internal consistency for the current sample (Cronbach’s *α* = 0.91).

##### Sociodemographic Characteristics

Data were collected on participants’ sex, age, educational attainment, sexual orientation, and relationship status and length.

#### Statistical Analysis Strategy

To undertake the analyses, we used the SPSS Statistics Version 21 package. We first investigated the relations among study variables using bivariate correlation analyses (see Table 1). Next, we secondly conducted two multiple regression analyses including (1) interpersonal sexual objectification and (2) partner objectification as predictor variables and sexual satisfaction and, unwanted sex rejection as the outcome variables to test Hypotheses 1 and 2, respectively. We then conducted two simple regression analyses including unwanted sex rejection as predictor and coercion victimization as the outcome variable to test Hypothesis 3 and, coercion victimization as predictor and sexual satisfaction as the outcome variable to test Hypothesis 4. Finally, two serial mediation analyses were run using PROCESS (Version 2; Model 6; Hayes, 2013) to examine the indirect effects of women’s perceived interpersonal objectification (Hypothesis 5a) and partner objectification (Hypothesis 5b) on sexual satisfaction based on rates of sexual rejection and coercion victimization. We included as the predictor (X) the degree to which women reported perceived interpersonal objectification (ISOS) or partner objectification (OBCS), sexual satisfaction as the criterion (Y), and unwanted sex rejection (M1) and coercion victimization (M2) as the mediating variables. Following Hayes’ (2013) procedures for testing indirect effects with serial mediators, bias-corrected confidence intervals for indirect associations were estimated based on 5,000 bootstrap samples. In these models, a CI that does not include 0 indicates a statistically meaningful association.

**Table 1:** Descriptive statistics and correlations among study variables for women.

Variables	*M*	SD	Correlations				
			1	2	3	4	5
1. Interpersonal objectification	2.56	0.60	–				
2. Partner objectification	3.30	1.10	0.33^**^	–			
3. Unwanted sex rejection	3.14	0.79	0.03	−0.32^**^	–		
4. Sexual coercion victimization	2.50	7.28	0.12	0.30^**^	−0.37^**^	–	
5. Sexual satisfaction	29.18	6.28	0.11	−0.20^*^	0.31^**^	−0.37^**^	–

*N = 138. Higher scores on continuous variables indicate greater standing on the variable (e.g., greater satisfaction)*.

* p < 0.05;

** p < 0.01;

*** *p < 0.001*.

### Results

Table 1 presents correlational and descriptive statistics for all variables. The correlations among the study variables were significant but also less than 0.70 indicating that there were no multicollinearity concerns.

Regression analyses were run to determine direct predictions. First, interpersonal sexual objectification did not predict sexual satisfaction, *b* = 1.18, *t* = 1.30, *p* = 0.20 (Hypothesis 1a). Conversely, perceived partner objectification was a significant predictor of women’s sexual satisfaction, *b* = −1.17, *t* = −2.30, *p* = 0.02, indicating that women who felt that their partners frequently surveyed their bodies were less likely to be sexually satisfied (Hypothesis 1b). Second, whereas interpersonal sexual objectification did not predict unwanted sex rejection, *b* = 0.04, *t* = 0.36, *p* = 0.72 (Hypothesis 2a), perceived partner objectification was a significant predictor of unwanted sex rejection, *b* = −0.24, *t* = −3.79, *p* < 0.001, showing that women who felt that their partners frequently objectified them were less likely to reject unwanted sex (Hypothesis 2b). Third, unwanted sex rejection was a significant predictor of coercion victimization, *b* = −3.34, *t* = −4.50, *p* < 0.001, indicating that women who were unable to reject unwanted sex were more likely to suffer coercion (Hypothesis 3). Finally, being a victim of coercion was a significant predictor of sexual satisfaction, *b* = −0.32, *t* = −4.52, *p* < 0.001; thus, women who were victims of coercion were less sexually satisfied.

#### Indirect Effect of Interpersonal Sexual Objectification and Perceived Partner Objectification on Women’s Sexual Satisfaction Based on Rates of Sexual Rejection and Sexual Coercion

Indirect effects can exist in the absence of a significant total effect (Zhao et al., 2010). Thus, to examine the indirect effects of women’s perceived interpersonal objectification (Hypothesis 5a) and partner objectification (Hypothesis 5b) on sexual satisfaction based on rates of sexual rejection and coercion victimization, two serial mediation analyses were run using PROCESS (Version 2; Model 6; Hayes, 2013).

**Figure 1:**
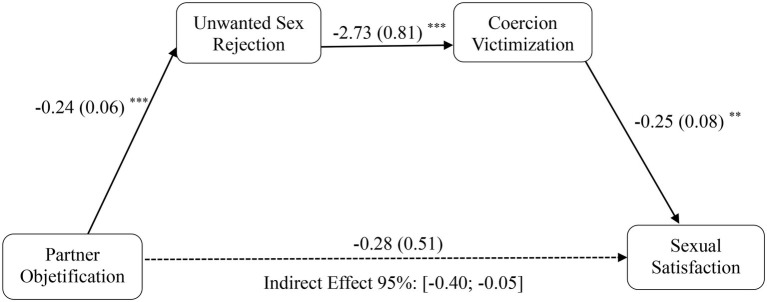
Serial mediation model depicting indirect effect of perceived partner-objectification and sexual satisfaction through unwanted sex rejection and coercion victimization, for women. Unstandardized beta coefficients reported, with standard errors within parentheses. In these models, a CI that does not include 0 indicates a statistically meaningful association. ^*^p < 0.05, ^**^p < 0.01, ^***^p < 0.001.

Regarding Hypothesis 5a, there was no evidence of an indirect effect of women’s perceived interpersonal objectification on sexual satisfaction based on rates of sexual rejection and coercion victimization [*b* = 0.04, SE = 0.11, 95% CI (−0.17, 0.27)]. Conversely, consistent with Hypothesis 5b, women’s perceived objectification by their partner was indirectly linked to lower sexual satisfaction *via* the effect of partner objectification on decreased unwanted sexual rejection capacity and increased coercion victimization [*b* = −0.16, SE = 0.08, 95% CI (−0.40, −0.05)]. Specifically, greater objectification from their partner was associated with decreased sexual rejection (Hypothesis 2b), which, in turn, was associated with increased coercion victimization (Hypothesis 3) and finally was related to higher sexual dissatisfaction (Hypothesis 4, controlling for sexual rejection and victimization; see Figure 1). Thus, Hypothesis 5b was fully supported. This pattern suggests that being objectified by only the partner (but not other men) is related to sexual dissatisfaction by undermining unwanted sex rejection capacity and, in turn, increasing the likelihood of suffering sexual coercion victimization.

## Study 2

### Method

#### Participants

A total of 156 men participated in the study. Of these, 13 were excluded because they did not identify as heterosexual and seven because they reported to be single and had never been in a sexual relationship with their current or former partner. The remaining 136 participants ranged in age from 17 to 34 years old (*M* = 23.02, SD = 3.77). In the sample, a total of 52.9% of the participants were dating, 9.6% were cohabiting, and 0.7% were married, whereas 36.8% were not involved in a romantic relationship. Approximately 48.9% of the participants completed high school and 51.1% were enrolled in a university degree program.

#### Procedure

Identical to the first study, the sample was obtained through an incidental sampling procedure in different university centers (e.g., libraries) in a Spanish city. Specifically, two evaluators informed the participants about the voluntary character of the study, the anonymity and confidentiality of their responses, and the study’s approximated duration. Additionally, single participants could answer the survey questions thinking about their most recent partner. After that, participants were asked to complete the questionnaire survey individually and voluntarily. Once participants accepted and provided informed consent, participants completed the study surveys embedded within a larger series of questionnaires. Like in the previous study, no reward was offered for participation and when study finished, participants were fully debriefed and thanked.

#### Measures

##### Interpersonal Objectification Perpetration

To evaluate the extent to which participants objectified women, participants completed the Interpersonal Sexual Objectification Scale-Perpetration (ISOS-P; adapted from the female Spanish version of Lozano et al., 2015 and translated from Gervais et al., 2018). This 15-item scale asks men to report on the frequency of which they engage in body evaluation (11 items; e.g., “How often have you made inappropriate sexual comments about someone’s body?”) and unwanted sexual advances (four items; e.g., “How often have you touched or fondled someone against her will?”), using a Likert-type scale ranging from 1 (never) to 5 (almost always). Participants’ responses were averaged, with higher scores indicating more frequent objectification of women. ISOS-P demonstrated good internal consistency for the current sample (Cronbach’s *α* = 0.86). Interpersonal Sexual Objectification Scale-Perpetration has adequate construct validity being positively associated with other measures assessing sexual objectification perpetration (Gervais et al., 2018).

##### Partner Objectification Perpetration

Men’s rated objectification of their partners was assessed using a modified version of the body surveillance subscale of the Objectified Body Consciousness Scale (OBCS-P; Moya-Garófano et al., 2017). Participants were asked to rate the extent to which they monitor their partner’s body (e.g., “I often think about whether the clothes my relationship partner is wearing make her look good”) on a Likert-type scale ranging from 1 (disagree strongly) to 7 (agree strongly), with a “not applicable” option. Not applicable responses were coded as missing data. Participants’ responses were averaged, with higher scores indicating greater objectification of their partner. The total partner objectification scale showed low reliability (= 0.43) and, as we did in Study 1, two items (5 and 6) were excluded, resulting in a six-item scale with higher but still low reliability (= 0.61), similar to the alphas found in previous studies (Cronbach’s *α* = 0.67; Zurbriggen et al., 2011).

##### Sexually Coercive Behaviors

An abbreviated version composed by two subscales of the Sexual Coercion in Intimate Relationships Scale (SCIRS; Shackelford and Goetz, 2004) was used to assess the frequency of sexual coercion of their intimate partner. Participants indicated whether they had perpetrated 10 specific acts of *commitment manipulation* (e.g., “I hinted that if my partner loved me, she would have sex with me”) and nine specific acts of *defection threat* (e.g., “I hinted that I would have sex with another woman if my partner did not have sex with me”). Participants answered using a six-point response scale: 0 (never has occurred), 1 (has occurred once in the last month), 2 (has occurred twice in the last month), 3 (has occurred between three and five times in the last month), 4 (has occurred between six and 10 times in the last month), and 5 (has occurred more than 11 times in the last month). Full-scale scores were calculated by summing response values for each item in the entire scale, so that higher numbers indicated higher sexual coercion perpetration. Because the item-level missing data can bias the sum score, we explored the percentage of the item-level missing data, and it was less than 5% of the participants, as Schafer (1999) suggested.

##### Sexual Satisfaction

The Global Measure of Sexual Satisfaction (GMSEX; Lawrance and Byers, 1995; Sánchez-Fuentes et al., 2015) was used to assess the participant sexual satisfaction with their partner. Respondents rated their sexual satisfaction on seven-point bipolar scales (very bad-very good; very unpleasant-very pleasant; very negative-very positive; very unsatisfying-very satisfying; and worthless-very valuable). Participants’ responses were summed (item-level missing data were less than 5%), with higher scores indicating greater sexual satisfaction. The Global Measure of Sexual Satisfaction demonstrated good internal consistency for the current sample (Cronbach’s *α* = 0.87).

##### Sociodemographic Characteristics

Data were collected on participants’ sex, age, educational attainment, sexual orientation, and relationship status and length.

#### Statistical Analysis Strategy

As in Study 1, we first investigated the relations among study variables using bivariate correlation analyses (see Table 2). Next, we secondly conducted two multiple regression analyses including (1) interpersonal sexual objectification and (2) partner objectification as predictor variables and sexual satisfaction and, coercive behaviors as the outcome variables, to test Hypotheses 1 and 2, respectively. We then conducted a simple regression analysis including coercive behavior as predictor and sexual satisfaction as the outcome variable to test Hypothesis 3. Finally, to examine the indirect effects of interpersonal objectification perpetration (Hypothesis 4a) and partner objectification perpetration (Hypothesis 4b) on sexual satisfaction after coercive behaviors, two mediation analyses were run using PROCESS (Model 4; Hayes, 2013). The degree to which men perpetrated interpersonal objectification (ISOS-P) or partner objectification (OBCS-P) was included as the predictor (X), sexual relationship satisfaction as the criterion (Y), and coercive behaviors (M) as the mediating variable. Following procedures recommended by Hayes (2013), indirect effects were tested using 5,000 bootstrapped samples; indirect effects are statistically significant if the 95% confidence interval does not include zero, and they can emerge even if direct effects are not significant (Zhao et al., 2010).

**Table 2:** Descriptive statistics and correlations among study variables for men.

Variables	*M*	SD	Correlations			
			1	2	3	4
1. Interpersonal objectification perpetration	1.98	0.45	–			
2. Partner objectification perpetration	3.42	1.15	0.20^*^	–		
3. Sexual coercive behaviors	3.49	11.07	0.50^**^	0.20^*^	–	
4. Sexual satisfaction	28.90	6.17	−0.32^**^	−0.19^*^	−0.38^**^	–

*N = 136. Higher scores on continuous variables indicate greater standing on the variable (e.g., greater satisfaction)*.

* p < 0.05;

** p < 0.01;

*** *p < 0.001*.

### Results

Table 2 presents correlational and descriptive statistics for all variables. The correlations among the study variables were significant but also less than 0.70 indicating that there were no multicollinearity concerns.

Then, regression analyses were run to determine direct predictions. First, both interpersonal sexual objectification, *b* = −4.39, *t* = −3.89, *p* < 0.001, (Hypothesis 1a) and partner-objectification perpetration, *b* = −1.02, *t* = −2.22, *p* = 0.03, (Hypothesis 1b) were significant predictors of men’s satisfaction, indicating that men who frequently surveyed bodies of their partner or other women were less likely be sexually satisfied. Second, both interpersonal sexual objectification, *b* = 12.15, *t* = 6.58, *p* < 0.001, (Hypothesis 2a) and partner objectification perpetration, *b* = 1.78, *t* = 2.32, *p* = 0.02, (Hypothesis 2b) were significant predictors of coercive behaviors, indicating that men who frequently objectified their partner or other women were also more likely to employ sexual coercion. Finally, coercive behaviors were a significant predictor of sexual satisfaction, *b* = −0.21, *t* = −4.65, *p* < 0.001; thus, men with a higher frequency of sexually coercive behaviors were less sexually satisfied (Hypothesis 3).

#### Indirect Effect of Interpersonal Sexual Objectification and Partner Objectification Perpetration on Men’s Sexual Satisfaction After Coercive Behaviors

Two mediation analyses were run using PROCESS (Model 4; Hayes, 2013) to examine the indirect effects of interpersonal objectification perpetration (Hypothesis 4a) and partner objectification perpetration (Hypothesis 4b) on sexual satisfaction after coercive behaviors.

**Figure 2:**
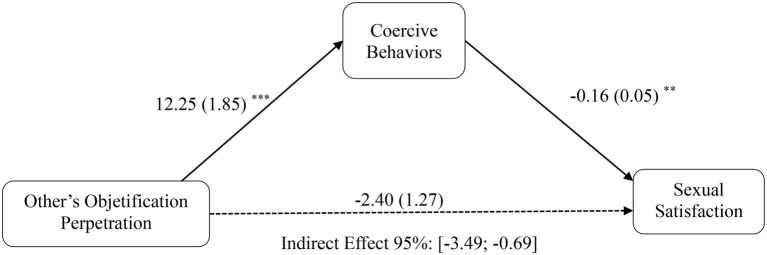
Mediation model depicting indirect effect of interpersonal objectification perpetration and sexual satisfaction through coercive behaviors, for men. Unstandardized beta coefficients reported, with standard errors within parentheses. In these models, a CI that does not include 0 indicates a statistically meaningful association. ^*^p < 0.05, ^**^p < 0.01, ***p < 0.001.

**Figure 3:**
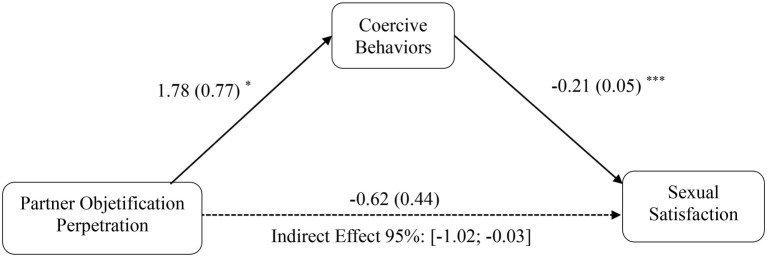
Mediation model depicting indirect effect of partner objectification perpetration and sexual satisfaction through coercive behaviors, for men. Unstandardized beta coefficients reported, with standard errors within parentheses. In these models, a CI that does not include 0 indicates a statistically meaningful association. ^*^p < 0.05, ^**^p < 0.01, ^***^p < 0.001.

Consistent with Hypothesis 4a, men’s interpersonal objectification perpetration was indirectly linked to lower sexual satisfaction *via* the effect of interpersonal objectification on increased coercive behaviors [*b* = −1.98, SE = 0.71, 95% CI (−3.49, −0.69); see Figure 2]. Moreover, consistent with Hypothesis 4b, partner objectification was also indirectly linked to lower sexual satisfaction *via* the effect of interpersonal objectification on increased coercive behaviors [*b* = −0.38, SE = 0.25, 95% CI (−1.02, −0.03); see Figure 3]. Specifically, a greater objectification perpetration of the partner (Hypothesis 2b) or other women (Hypothesis 2a) was associated with increased sexually coercive behaviors, which finally were related to higher sexual dissatisfaction (Hypothesis 3, controlling for coercive behaviors). Thus, Hypotheses 4a and 4b were fully supported.

## Discussion

The purpose of the present work was to examine the detrimental effect of sexual objectification victimization and perpetration on sexual satisfaction and test the mediational effect of sexual coercion in such relationship. Because sexual objectification is a gendered phenomenon where women are commonly the victims and men are the perpetrators (Strelan and Hargreaves, 2005), the current set of studies assumes a gender perspective. The first study examined how women’s perception of sexual objectification (in their daily life and in their romantic relationships) is indirectly related to sexual dissatisfaction because of their lower capacity to reject sexual approaches and consequently higher rate of sexual coercion victimization. Secondly, we examined how men’s perpetration of sexual objectification and partner objectification is indirectly linked to lower sexual satisfaction after increased sexual coercion perpetration. These results extend and conceptually replicate Ramsey and Hoyt’s (2015, p. 167) findings “showing some support for a relationship between partner objectification with sexual pressure and coercion in heterosexual romantic relationships” a conceptual replication crucial for theoretical development (Earp and Trafimow, 2015). Moreover, the results presented here expand the findings of Ramsey and Hoyt (2015) showing that generally, objectification perpetration had the same negative outcomes as partner objectification, whereas from a female victim’s perspective, distinguishing who committed sexual objectification is important.

### Women’s Experienced Objectification and Sexual Satisfaction Mediated by Lower Sexual Rejection and Higher Sexual Coercion Rates

In Study 1, our main finding reveals in women an indirect association between perceiving sexual objectification by their partner and the lower relationship satisfaction because of the women’s lower capacity for sexual rejection as well as a higher level of sexual coercion victimization. The relation between partner objectification and lower sexual satisfaction is explained because male partner dehumanization involved in the focus on appearance leads to a negative female body image, which affects the sexual satisfaction in an intimate domain (Sáez et al., 2019). This result is built on previous studies that showed how feeling objectified by their partner decreases female sexual desire (Ramsey and Hoyt, 2015), which turns into lower sexual satisfaction. Interestingly, we did not find a relationship between sexual objectification experiences and sexual dissatisfaction. This pattern of results means that even for women who are targets of objectification once every 2 days (Holland et al., 2017), being objectified in an intimate domain is the variable positively related to sexual dissatisfaction, partially supporting Hypothesis 1.

In addition, results showed that being a target of partner objectification is associated to a lower capacity to refuse unwanted sex (Hypothesis 2 was partially supported). This result agrees with previous studies that identified the potential mechanism that explains why sexual objectification leads to higher rates of sexual victimization (Franz et al., 2016). However, general sexual objectification perpetration was not found to decrease women’s ability to reject a sexual advance, which might be because there is not a direct relationship between objectification and sexual assertiveness, but there is an indirect effect because of self-objectification (Franz et al., 2016). It might be that just women who treat themselves as sexual objects for male sexual satisfaction reject less sexual advances when they are not willing to consent to such an approach.

Although it was not hypothesized, we found a positive relation between general sexual objectification victimization and the extent women perceive partner objectification, which might be due to the overlap between the two measures. Those women who perceive body monitoring by their partner (greater partner objectification) will experience a higher number of sexual objectification experiences because their partner’s objectifying lens will increase his number of sexual objectification behaviors. For example, a woman who perceives her partner as chronically focused on her appearance is going to perceive that generally, she is more a target of stares and comments about her body because in part, those behaviors would come from her interpersonal experiences with her partner. The interpersonal sexual objectification experiences scale does not distinguish among different perpetrators of those behaviors, and our findings support the idea that some negative consequences of sexual objectification depend of the perpetrator of such objectification. We encourage researchers in the sexual objectification field to explore the different outcomes of sexually objectified interactions depending on the relationship with the perceiver. Our findings support the belief that some specific life areas (as sexual intimacy) are especially sensitive depending on the perpetrator (partner or stranger) of sexual objectification.

Moreover, the results support the second hypothesis. Only perceived partner surveillance (but not general objectification) is related to a damaged capacity to reject unwanted sexual advances, and this finding is complemented by previous studies that show that self-surveillance and body shame are mediators of the link between partner objectification and refusal of sexual advances (Ramsey and Hoyt, 2015). Women who believe that their partner is exclusively focused on their appearance instead of being concerned about their feelings and thoughts might perceive less self-efficacy for stopping an unwanted sexual advance. This low self-efficacy might hinder their assertiveness for expressing what they want and what they do not want to do sexually, which, in line with previous studies, leads to greater sexual victimization (Ramsey and Hoyt, 2015). The current work extends previous research showing that sexual coercion victimization as a result of the lower female capacity to refuse sex with partners chronically focused on their appearance may turn into lower female sexual satisfaction (consistent with Hypotheses 3 and 4).

Lastly, Hypothesis 5 was partially supported with partner objectification (but not general objectification victimization), the unique predictor of sexual satisfaction because of a lower capacity to reject sexual advances and a higher rate of sexual coercion victimization. In summary, the serial mediational analysis allowed us to conclude that women’s perception of being a sexual object in their partner’s eyes is linked to higher female censure of their desires (or lack of them) previous to sexual intercourse, which in turn, is associated to higher rates of sexual victimization and is incompatible with sexual satisfaction. A couple’s counseling therapist might consider this result extremely interesting for increasing the woman’s sexual satisfaction by making her male partner focus on her feelings and desires instead of her appearance as well as prompting the female partner to put her desires ahead of the male partner’s.

### Men’s Perpetration of Objectification and Sexual Satisfaction Mediated by Sexual Coercion

In Study 2, both objectification toward women in general and partner objectification show similar pattern of outcomes. These findings, according with Sáez et al. (2019), showed that general objectification is closely linked to partner appearance surveillance. Specifically, our results support the first hypothesis: interpersonal objectification perpetration and partner objectification was directly related to male sexual satisfaction. This result is according to previous studies on objectification literature that have showed how partner objectification is negatively related to sexual satisfaction (Zurbriggen et al., 2011; Sáez et al., 2019) because sexual objectification far from promote male sexual satisfaction and prevent them to have satisfactory relations with women in an intimate level. Those men who perceive women through an objectifying lens endorse sexual reductionism (i.e., evaluation and perception of women based on their bodies and on their physical sexual appearance), which makes it difficult to have satisfactory sexual intimacy with real women (Brooks, 1995). In addition, the correlational results presented in the heterosexual male study showed the significance in the relationship between partner objectification and women’s general objectification. This result replicates the finding of Sáez et al. (2019), supporting the idea that men who rely on an objectifying lens when perceiving women are more likely to perceive their romantic partners through the same lens.

Moreover, our findings support Hypotheses 2a and 2b, revealing that men’s perpetration of sexual objectification and men’s partner objectification are linked to more sexually coercive behaviors toward their partner or former partner. We propose understanding female sexual objectification as a behavioral continuum from subtle to extreme behaviors, in which making sexual commentaries about women’s bodies would be at the lowest part of the continuum, and sexual aggression or sexual coercion must be at the other extreme of the same continuum. This idea could explain the high correlation between sexual objectification perpetration (body evaluation and unwanted sexual advances) and sexual coercion perpetration, and moreover, it is congruent with previous studies that have already showed the association between sexual and sexual violence (Rudman and Mescher, 2012; Gervais et al., 2014). Those results together allow us to conclude that men’s objectifying focus on a specific woman (their partner) or sexually objectifying behaviors toward women in general are ones of the predictors of sexually abusive behaviors toward them. Psychologists working on sexual violence prevention should consider these results interesting because increasing the male perception of women as fully human, with a focus on women’s character regardless of their appearance, might be an effective preventive strategy. However, because of the correlational nature of the present study, results may be interpreted the other way around: that is, committing sexual violence toward women leads those men to perceive women as violable objects, which leads to greater focus on their appearance. However, previous experimental studies support the idea that implicit sexual objectification triggers sexual violence toward women (Rudman and Mescher, 2012).

Moreover, the current research fills a gap in the literature exploring the interpersonal consequences of sexual coercion for men, showing that sexual coercion is going to lead to lower male sexual satisfaction (consistent with Hypothesis 3). This remarkable result means that while men coerce and pressure their female partners to fulfill their masculine sexual desires, such coercive behaviors could be linked to detrimental effects for their sexual satisfaction.

Lastly, the significance of the mediational models tested suggests that in men, a higher tendency to perpetrate objectifying behaviors as well as chronically focused on their partner’s appearance is associated to a lower sexual satisfaction because of the use of sexually coercive strategies to have sex with their partners, which supports Hypotheses 4a and 4b. A possible explanation for those indirect effects is the dehumanization process involved in the perception and treatment of women as objects (Loughnan et al., 2010). The association found between male sexual objectification (toward women in general and toward their partner) and sexual coercion agrees with classical feminist philosophers (Nussbaum, 1999) who support the idea that objectification involves the belief of women’s value as the extent to which they can sexually satisfy men (instrumentality) and if they are not sexually useful, they might become targets of violence (violability).

### Limitations and Future Research Directions

The present research provides evidence of objectification’s relevance for both women and men’s sexual satisfaction and the mechanisms underlying these associations. Yet there are some limitations that also offer other valuable opportunities for future research.

First, the sample of our research is composed of a young population, most of whom were involved in short-term relationships or alternatively, completed the questionnaire based on a previous relationship, hindering replication of past findings and generalization of the results to the general population. Future studies using a larger sample composed of individuals in long-term committed relationships should corroborate our findings in more detail.

Second, the strength of the present research is studying objectification within relationship contexts using a sample of women and men involved in a relationship. However, (1) our studies focused solely on one member of the couple and (2) according to previous literature which revealed that women are usually the objectification targets (Fredrickson and Roberts, 1997), women have been considered objectification recipients and men perpetrators. Examining couples’ perceptions and dynamics simultaneously in different types of relationships (e.g., mixed-sex and same-sex) is a gap that needs to be addressed for untangling how objectification influences people’s interactions. For example, a dyadic study in which the partner communication—affected by gender roles (Alonso-Ferres et al., 2019)—can be observed and analyzed, could improve the current knowledge of how objectification phenomenon works within romantic relationship. Likewise, a study of experience sampling could also help to explain the specific circumstances underlying objectification, thus providing strong external validity of the results. Moreover, in order to test that effects found are framed in sexual objectification phenomenon, future studies should confirm that victimization effects are exclusive for female partners of heterosexual relationships and the perpetrators effects are significant only for male partners of heterosexual relationships.

Third, the correlational and cross-sectional nature of our study prevents us from establishing any causal relationships between variables. For example, sexually coercive men are more prone to objectifying women, so sexual objectification can be thought as a trait of people who are more prone to sexual coercion or women with lower sexual assertiveness are inclined to establish relationship with objectifying perpetrators. In order to handle these limitations, it could be interesting that the future research use experimental studies. Future research could also go a step further by studying additional variables related to partner objectification and coercion. For example, subscribing Ramsey and Hoyt (2015), we encourage researchers to explore the role that power plays in romantic relationships where objectification occurs. Power theories argue that powerful people possess and control valuable resources and do not need others to pursue their desired outcomes, and thus, they behave selfishly (Keltner et al., 2003). In contrast, those who lack power often rely on others for support and assistance in facing the challenges of their life and, in turn, tend to adopt submissive behaviors. Consequently, as Gruenfeld et al. (2008) proposed, having power could enhance objectification and partner objectification as well as sexually coercive behaviors, whereas lacking power could be related to the incapacity to reject unwanted sex after being objectified by a partner. A variable other than the direct or behavioral effects of interpersonal or partner objectification that could be added into future studies could be the emotions and cognitions (inferences) women feel after being objectified, specifically because a previous work has shown the negative association between body surveillance and dissatisfaction because of body shame (Sun, 2018).

The fourth limitation is related to the low reliability of the Partner Objectification Scale in both studies (victimization and perpetration). Although previous studies using these measures have found similar low reliability (Zurbriggen et al., 2011; Ramsey et al., 2017), it can jeopardize results and conclusion and it makes necessary steps to replicate the conclusion using different measures. Future research should continue developing more appropriate scales for assessing partner objectification in their perpetration and victimization versions.

Finally, it is essential to continue looking into alternative possible relational and personal causes and consequences of interpersonal and especially partner objectification (such as an individual’s well-being or health) because it could be an important way to exert and explain interpersonal violence that many people who are part of a couple suffer throughout their lives. Moreover and related to partner objectification perpetration, it is unclear why men sexually objectify their partners despite the negative outcomes linked with objectification perpetration (lower sexual satisfaction). We encourage future studies to explore motivations that endure sexual objectification phenomenon in romantic contexts as well as a more comprehensive theoretical articulation of the objectification perpetration that allows explaining empirical evidence that has exceeded the original assumptions of objectification theory.

## Conclusions and Practical Implications

Objectification Theory explores the consequences that women suffer for living on Western culture, where they are treated as sexual objects. However, previous research has showed both cross-cultural (e.g., Loughnan et al., 2015) and within-culture differences (e.g., Gervais et al., 2015) on this phenomenon. Specifically, sexual objectification is higher on Western cultures compared to Eastern cultures, and on Western culture, not all men treat women as sex objects in the same extent, being attitudinal (Cikara et al., 2011) and individual variables (social comparison or cultural orientation, Gervais et al., 2015) crucial for predicting sexual objectification perpetration within the same culture. The present study through two within-culture studies aims to explore the individual variables related to (perpetrate or suffer) higher sexual objectification and its negative outcomes.

The present research is essential for understanding the consequences that partner objectification has for female victims and male perpetrators of sexual assault and improving the dynamics within heterosexual relationships. The findings across the two studies show that women objectified by their partner usually have lower capacity to refuse sex, which leads to a higher probability of suffering sexual coercion and, in turn, is associated to a lower sexual satisfaction in their relationships. Furthermore, objectification also has consequences for the men who perpetrate it, so that perpetrators (both general and partner) may be more likely to become sexual coercion offenders, which is also linked to a decrease in their sexual satisfaction. Our study supports the findings of Rudman and Mescher (2012) and Gervais et al. (2014), showing that sexual objectification sets the stage for sexual violence. It is especially important for risk assessment of sexual offenders, being objectification a risk factor which needs to be identified (Bonta and Andrews, 2007) and female objectification a key element in the cognitive treatment of sex offenders. Moreover, prevention programs must emphasize the identification of inappropriate sexual behaviors in young men when their gender role is beginning to take shape (Butchart et al., 2010). Interventions should pay attention to the phenomenon of objectification, which has consequences for both victims and perpetrators, with the intention of fully understanding it and working on it to improve women’s overall welfare and reduce objectification perpetration. Finally, increasing sexual assertiveness or discouraging women from entering or remaining in coercive relationships may help to prevent the negative consequences of objectification. It is also necessary to consider different types of sexual victimization (sexual coercion and sexual aggression) and different perpetrators (partners, acquaintances, or strangers) as separate phenomenon in order to get a better understanding of and ultimately prevent sexual victimization.

## Data Availability Statement

The datasets generated for this study are available on request to the corresponding author.

## Ethics Statement

This research was carried out in accordance with the recommendations of Human Research Ethics Committee of the University of Granada. Participants provided informed consent in accordance with the 1964 Declaration of Helsinki.

## Author Contributions

Each author has made substantial contributions to the conception or design of the work. GS, MA-F, MG-M, IV-S, and FE jointly drafted the content of the paper, where GS was the leader. MA-F and MG-M collected the data of Study 1 and 2, whereas GS, MA-F, and MG-M analyzed and interpreted the data of Study 1 and 2. All authors provided approval for publication of the content and agreed to be accountable for all aspects of the work in ensuring that questions related to the accuracy or integrity of any part of the work are appropriately investigated and resolved.

### Conflict of Interest

The authors declare that the research was conducted in the absence of any commercial or financial relationships that could be construed as a potential conflict of interest.
